# Corneal epithelial remodeling after femtosecond laser-assisted in situ keratomileusis combined with intraoperative accelerated corneal collagen crosslinking for myopia: a retrospective study

**DOI:** 10.1186/s12886-022-02570-0

**Published:** 2022-08-20

**Authors:** Junjie Piao, Shen Wang, Ye Tao, Yue Hua Zhou, Ying Li

**Affiliations:** 1 Department of Ophthalmology, Peking Union Medical College Hospital, Chinese Academy of Medical Sciences and Peking Union Medical College, No.1 Shuai fu yuan, Dong Cheng District, Beijing, 100730 China; 2 Department of Medical Science Research Center, Translational Medicine Center, Peking Union Medical College Hospital, Chinese Academy of Medical Sciences and Peking Union Medical College, Beijing, 100730 China; 3 Beijing Vision Optometry, Beijing, 100191 China; 4 College of Ophthalmology, Chengdu University of Traditional Chinese Medicine, Chengdu, 610075 Sichuan China

**Keywords:** LASIK Xtra, Myopia, Corneal thickness, Keratometry, Ocular response analyzer

## Abstract

**Background:**

This study analyzed regional corneal thickness remodeling, biomechanical properties, and visual outcomes after femtosecond laser-assisted in situ keratomileusis combined with intraoperative accelerated corneal collagen crosslinking (LASIK Xtra) for myopia.

**Methods:**

This retrospective study analyzed 21 consecutive patients (18 women, three men; 42 eyes) who were treated with LASIK Xtra. All treatments were performed with ultraviolet-A (energy, 2.7 J/cm^2^; irradiance, 30 mW/cm^2^), using continuous (90 s) illumination. Postoperative values of corneal biometrics and visual outcomes were compared with preoperative values. Corneal thickness changes were evaluated using anterior segment optical coherence tomography. All patients were followed up for 12-month postoperatively. Preoperative and postoperative data were compared statistically using the paired t-test for normally distributed parameters and the Wilcoxon rank-sum test and Friedman analysis of variance with Bonferroni correction for non-normally distributed data.

**Results:**

Uncorrected distance visual acuity (UDVA) significantly improved at 6-month after surgery (*P* < 0.001). The central and inner regional corneal epithelial thickness significantly increased after LASIK Xtra (*P* < 0.05 for all), while the peripheral corneal epithelial thickness remained stable at 12-month after surgery. There was also a statistically significant decreased in the stromal thickness at most locations (*P* < 0.05 for all), except in the outer superior and outer superior-temporal regions.

**Conclusions:**

LASIK Xtra provided improvement in UDVA, corneal curvature, and corneal biomechanical stability. Because the results of this retrospective study results depended on the cohort members’ past information, it was inferred and confirmed that regular corneal thickness remodeling occurred after treatment.

## Background

Myopia has emerged as a major health issue in Asia, because of the increased use of electronic devices and modern living habits. The increased importance of esthetics amongst the general population and occupational requirements of first responders, athletes, those who work in the military and the media, and other professions, has resulted in an increase in contact lens use; however, there is a risk of long-term discomfort and infection associated with contact lens use [1]. This has increased the demand for surgical vision correction procedures for conditions such as myopia, hyperopia, and irregular astigmatism. However, iatrogenic keratectasia has emerged as a serious complication of laser vision correction surgery [2]. Owning to advancements in diagnosis and femtosecond laser platforms, the incidence of iatrogenic keratectasia has decreased from 0.66 to 0.021% [3, 4]. Risk is associated with the amount of tissue altered and therefore, high myopia, unevenly distributed corneal thickness, abnormal corneal topography, hyperopic treatments, weakened corneal biomechanical properties, and thin predicted residual corneal thickness are risk factors [2, 5].

Corneal collagen crosslinking (CXL) uses the combined action of riboflavin and ultraviolet-A (UVA) to cause photopolymerization of corneal collagen fibers, enhancing corneal biomechanical strength through the formation of new covalent bonds. It is a safe and effective therapeutic technique for halting the progression of keratoconus and iatrogenic keratectasia. Consequently, femtosecond laser-assisted in situ keratomileusis (LASIK) procedures combined with intraoperative accelerated corneal collagen crosslinking (LASIK Xtra) were developed [6, 7]. LASIK Xtra is associated with safety and efficacy similar to that of conventional LASIK procedures [4, 8].

The VISX Star S4 excimer laser system’s ablation software has a tendency toward central islands (CI) formation [9], and the effect of CI formation on visual rehabilitation, safety, and stability after LASIK Xtra (energy, 2.7 J/cm^2^; irradiance, 30 mW/cm^2^) has not been fully elucidated. In 2014, an anti-CI formation software was used with the VISX Star S4 excimer laser, which significantly decreased the rate of CI formation and improved postoperative best-corrected distance visual acuity [9, 10]. The differences in epithelial profiles can mask measures of corneal topography, such as corneal stress concentration directions between the optical zone and untreated ablation zone [10, 11].

LASIK Xtra has significantly reduced the epithelial increase [12] and corneal haze [13] compared to LASIK. The corneal anterior stromal fibroblast activity not only has an effect on corneal haze, but also on corneal elasticity. Moreover, regional differences in corneal epithelial healing occur after corneal refractive surgery with the VISX Star S4 excimer laser. We hypothesized that the corneal epithelial profiles would be regular and remain stable after LASIK Xtra following VISX Star S4 excimer laser combined with accelerated superficial CXL. This retrospective study aimed to clinically evaluate corneal epithelial and stromal remodeling after LASIK Xtra.

## Methods

This retrospective study analyzed 21 consecutive patients (42 eyes) diagnosed with moderate to high myopia at the Department of Ophthalmology, Peking Union Medical College Hospital, Beijing, China, between February 2017 and October 2018. The study protocol followed the guidelines of the Declaration of Helsinki and the Institutional Review Board for Human Studies and was approved by the Peking Union Medical College Hospital Institutional Ethics Committee (S-K1768-1). Written informed consent was obtained from all the patients before the study was initiated.

Inclusion criteria for the study were as follows: borderline corneal topography (defined as 1 diopter [D] or greater inferior steepening in some areas but an inferior-superior value of less than 1.4 D or unevenly distributed corneal thickness) or more than -6 D spherical equivalent with residual stromal bed thickness less than 250 μm [14], and the potential to improve postoperative refractive stability regarding myopic regression, and therefore, a lower risk of iatrogenic keratectasia. All patients were aged > 18 years and had no other ocular pathologic signs (such as ocular surface infection or allergy).

Exclusion criteria included history of ocular surgery or conditions, such as diabetes, autoimmune or endocrine pathologies, dry eye symptoms, insufficient follow-up, re-treatment of accelerated CXL, and pregnancy or lactation.

### Surgical procedures

All procedures were performed by an experienced surgeon (Y.L.). After topical anesthesia with 0.5% proparacaine (Alcaine, Alcon-Couvreur; Puurs-Sint-Amands, Belgium, USP), a corneal flap was created using VisuMax (Carl Zeiss Meditec AG, Jena, Germany) femtosecond laser platforms. Using a superior hinge, an intended flap diameter of 8.5 mm, and a flap thickness of 90–95 μm, corneal ablation was performed with the VISX S4IR excimer laser (Abbott Medical Optics, Santa Ana, CA, USA). Then, the corneal bed was saturated with a solution of 0.22% dextran-free riboflavin ophthalmic solution in normal saline (Vibex Xtra™, Avedro, Waltham, MA, USA), which was deposited at the center of the cornea and allowed to soak for 90 s. After complete soaking of the riboflavin, the solution was rinsed from the eye with a 0.9% balanced saline solution. Next, UVA energy was applied at 2.7 J/cm^2^ with an irradiance of 30 mW/cm^2^ to the eye that underwent continuous (90 s) light illumination for LASIK Xtra (KXL system; Avedro, Inc., Waltham, MA, USA) (Table 1). Finally, the eye was rinsed again with a 0.9% balanced saline solution, and a bandage contact lens (PureVision; Bausch & Lomb, Rochester, NY, USA) was applied to the cornea until complete re-epithelialization was achieved.

**Table 1:** LASIK Xtra methods

Parameter	LASIK Xtra
Treatment target	Myopia
Fluence (total) (J/cm^2^)	2.7
Soak time and interval (s)	90
Intensity (mW)	30
Treatment time (s)	90
Epithelium status	Off
Chromophore	Riboflavin solution in normal saline (Vibex Xtra™, Avedro, Waltham, MA, USA)
Chromophore carrier	Dextran-free
Chromophore osmolarity	Iso-Osmolar
Chromophore concentration	0.22%
Light source	UV-A light (KXL system; Avedro, Inc., Waltham, MA, USA)
Irradiation mode (interval)	Continuous
Protocol modifications	None

LASIK Xtra = 30*90 (femtosecond laser-assisted in situ keratomileusis combined with accelerated corneal collagen crosslinking), *J* Joule, *s* Second, *mW* Milliwatt, *UV-A* Ultraviolet-A

After surgery, all patients were prescribed topical 0.5% levofloxacin 4 times daily for 2 weeks, 0.5% loteprednol etabonate (Lotemax; Bausch & Lomb, Tampa, FL, USA), and preservative-free artificial tears (Hycosan; Hylo-Comod, Ursapharm Arzneimittel, Saarbruecken, Germany) 4 times daily for 1 month, and 0.2% carbomer eye gel (Liposic; Bausch & Lomb, Brunsbütteler Damm, Berlin, Germany) once daily at night for 1 month.

### Outcome measures

Examinations were performed preoperatively, and postoperatively at 1 and 3 days; 1 week; and 1, 3, 6, and 12 months. Slit-lamp examination, best spectacle-corrected visual acuity with and without a pinhole, uncorrected distance visual acuity (UDVA), corrected distance visual acuity (CDVA) using a logarithm of the minimum angle of resolution (logMAR) chart with tumbling E, corneal topography (TMS-4 N; TOMEY, Erlangen, Germany), dual Scheimpflug imaging (Gallilei; Ziemer Ophthalmology, Port, Switzerland), and ultrasonic pachymetry (TOMEY Ltd., Aichi, Japan) of the central cornea were performed. Goldmann applanation tonometry and an ocular response analyzer (Reichert Technologies; Depew, NY, USA) were used to measure intraocular pressure (IOP) and corneal biomechanical properties. The corneal demarcation line and the corneal epithelial and stromal thicknesses were measured and evaluated using anterior segment optical coherence tomography (AS-OCT) (Optovue RTVue XR, Optovue; Fremont, CA, USA). Corneal epithelial and stromal thickness profiles were obtained at the thinnest part of the central cornea using 16 peripheral measurements on the corneal vertex, and measurements and statistical analyses of the central 6-mm zone (inner areas, 3-mm zone of corneal vertex; outer areas, 6-mm zone of corneal vertex) were performed [15]. For ultrasound pachymetry, the average measurements of the corneal thickness values were chosen (each single measurement represented the mean of 5 consecutive measurements). Postoperative evaluation included UDVA, corneal biomechanical parameters, corneal topography, and corneal thickness profiles (epithelia and stroma) using AS-OCT.

### Statistical analyses

Data regarding all the evaluated parameters were recorded in a Microsoft Excel spreadsheet (Microsoft; Redmond, WA, USA), and statistical analysis was performed using SPSS for Mac (version 25.0; IBM Corp., Armonk, New York, USA). Data normality was tested using the Shapiro–Wilk test, and a paired t-test was performed to analyze changes between preoperative and postoperative data. If the data were not normally distributed, the Wilcoxon rank-sum test was performed to analyze changes between preoperative and postoperative data. Friedman analysis of variance with the Bonferroni correction was applied for repeated parameter measurements at the 12-month follow-up, in case data did not show a normal distribution. Statistical significance was set at *P* < 0.05.

## Results

Forty-two consecutive eyes underwent bilateral LASIK Xtra. This study included 18 women (86%) and three men (14%). The mean age was 25.76 ± 5.35 years, and the mean preoperative UDVA and CDVA were 1.38 ± 0.23 logMAR and -0.01 ± 0.03 logMAR, respectively. The preoperative manifest refractive spherical equivalent was -7.28 ± 2.35 D. Demographic details and refractive outcomes are presented in Table 2.

**Table 2:** Preoperative patient demographic data

Parameter	LASIK Xtra
Eyes/Patients (n)	42/21
Age (y)	25.76 ± 5.35
Sex (M/F)	3/18
MRSE (D)	-7.28 ± 2.35
Ks (D)	44.68 ± 1.51
AvgK (D)	44.01 ± 1.44
Cyl (D)	1.34 ± 0.85
UDVA (logMAR)	1.38 ± 0.23
CDVA (logMAR)	-0.01 ± 0.03

*LASIK Xtra* Femtosecond laser-assisted in situ keratomileusis combined with intraoperative accelerated corneal collagen crosslinking, *y* Years, *MRSE* Manifest refraction spherical equivalent, *Ks* Steepest keratometry, *AvgK* Front average keratometry, *Cyl* Keratometric astigmatism, *D* Diopters, *UDVA* Uncorrected distance visual acuity, *CDVA* Corrected distance visual acuity, *logMAR* logarithm of the minimum angle of resolution

### Epithelial and stromal remodeling

**Fig.1:**
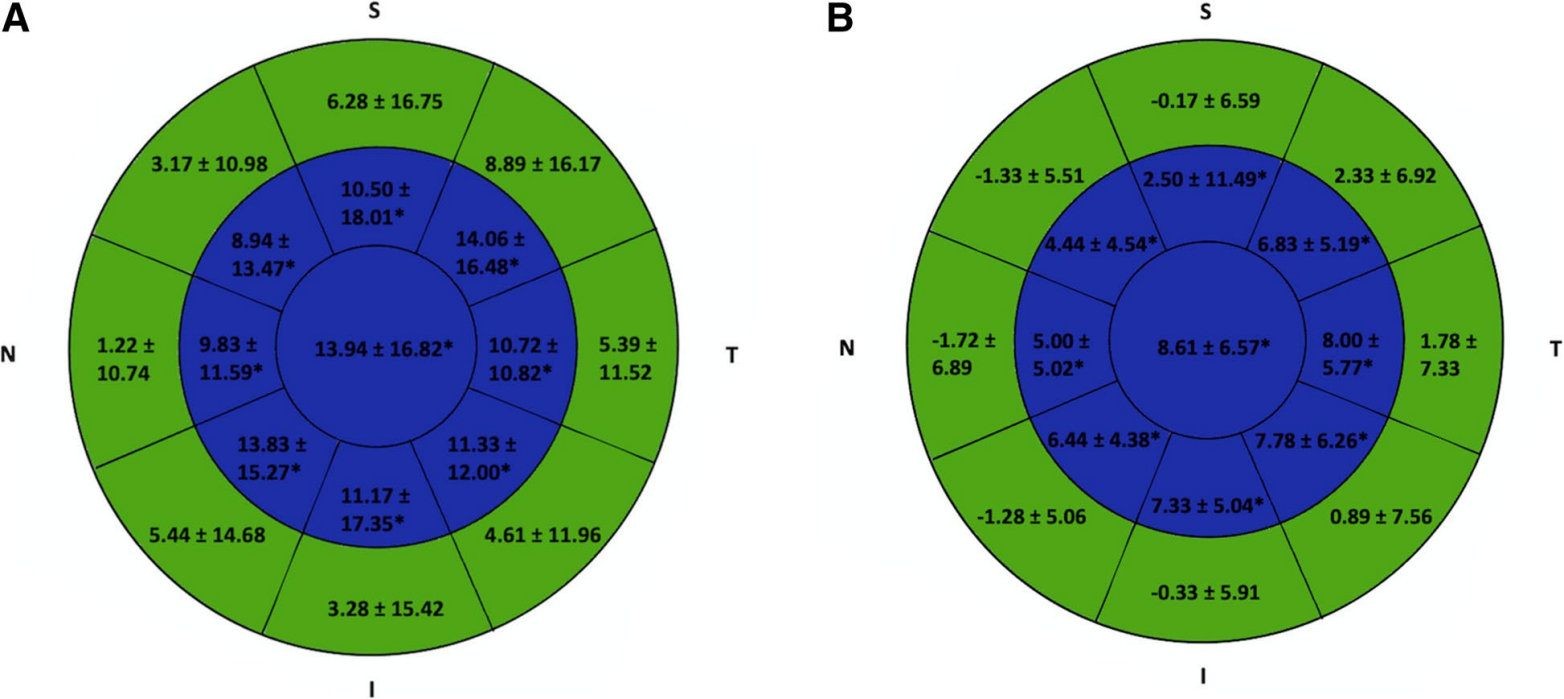
Mean changes in epithelial thickness distribution after femtosecond laser-assisted in situ keratomileusis combined with intraoperative accelerated corneal collagen crosslinking during the 12-month follow-up (**A**. 6-month postoperatively; **B**. 12-month postoperatively). Values in blue indicate a significant change after surgery (*P* < 0.05). S: superior; I: inferior; T: temporal; N: nasal

The preoperative and postoperative corneal epithelial and stromal thickness changes after 12-month are summarized in Tables 3 and 4. There was a statistically significant thickened in the central and inner regional corneal epithelial thickness due to remodeling during the 12-month follow-up (*P* < 0.05 for all). The outer regional epithelium was thickened at 6-month postoperatively and repopulated at 12-month postoperatively (*P* > 0.05 for all). There was a statistically significant thinning in the stromal thickness profiles in most locations (*P* < 0.05 for all). The comparison of the differences between the preoperative and postoperative values are shown in Fig. 1A and B.

**Table 3:** Mean corneal stromal thickness changes after LASIK Xtra

	Mean ± SD			
Parameter	Pre-op	6-month Post-op	12-month Post-op	*P* value
Central (μm)	496.22 ± 22.94	424.39 ± 27.03*	429.11 ± 26.04*	< 0.001
Inner Inferior (μm)	507.72 ± 23.32	461.17 ± 21.49*	464.83 ± 23.92*	< 0.001
Inner Inferior-nasal (μm)	512.44 ± 23.98	464.28 ± 26.22*	448.11 ± 94.66*	< 0.001
Inner Nasal (μm)	518.22 ± 24.22	469.83 ± 29.03*	475.50 ± 29.55*	< 0.001
Inner Superior-nasal (μm)	528.56 ± 24.44	483.00 ± 28.54*	485.50 ± 29.70*	< 0.001
Inner Superior (μm)	530.78 ± 24.29	489.50 ± 28.47*	492.56 ± 25.93*	< 0.001
Inner Superior-temporal (μm)	520.22 ± 23.26	477.11 ± 27.47*	478.56 ± 25.10*	< 0.001
Inner Temporal (μm)	506.94 ± 23.62	461.67 ± 25.48*	462.83 ± 23.70*	< 0.001
Inner Inferior-temporal (μm)	504.11 ± 23.09	458.83 ± 21.50*	460.17 ± 22.51*	< 0.001
Outer Inferior (μm)	536.57 ± 28.09	512.17 ± 18.80*	511.00 ± 25.84*	0.016
Outer Inferior-nasal (μm)	533.22 ± 24.41	516.83 ± 25.77*	516.44 ± 30.19*	0.023
Outer Nasal (μm)	538.44 ± 24.84	525.67 ± 28.24*	525.11 ± 36.98	0.090
Outer Superior-nasal (μm)	545.72 ± 24.70	543.83 ± 24.45	535.33 ± 44.19	0.114
Outer Superior (μm)	560.67 ± 25.75	554.72 ± 25.97	549.22 ± 33.33	0.220
Outer Superior-temporal (μm)	568.39 ± 23.29	531.83 ± 30.09	528.22 ± 33.49	0.100
Outer Temporal (μm)	528.28 ± 23.27	504.56 ± 30.25*	502.94 ± 31.22	0.050
Outer Inferior-temporal (μm)	525.44 ± 23.12	504.39 ± 20.27*	507.39 ± 30.98*	0.023

*LASIK Xtra* Femtosecond laser-assisted in situ keratomileusis combined with intraoperative accelerated corneal collagen crosslinking, *SD* Standard deviations, *μm* Micrometer, *Pre-op* Preoperative, *Post-op* Postoperative

^*^P value was statistically significant differences between postoperative and baseline

**Table 4:** Mean corneal epithelial thickness changes after LASIK Xtra

	Mean ± SD			
Parameter	Pre-op	6-month Post-op	12-month Post-op	*P* value
Central (μm)	53.11 ± 3.20	67.06 ± 16.90*	61.72 ± 5.51*	< 0.001
Inner Inferior (μm)	53.83 ± 3.24	65.00 ± 17.98*	61.17 ± 4.68*	< 0.001
Inner Inferior-nasal (μm)	53.83 ± 3.09	67.67 ± 15.51*	60.28 ± 4.16*	< 0.001
Inner Nasal (μm)	53.11 ± 2.74	62.94 ± 11.84*	58.11 ± 4.74*	< 0.001
Inner Superior-nasal (μm)	53.06 ± 3.06	62.00 ± 14.59*	57.50 ± 4.67*	< 0.001
Inner Superior (μm)	52.61 ± 3.13	63.11 ± 19.35*	55.11 ± 12.09*	0.001
Inner Superior-temporal (μm)	52.28 ± 2.91	66.33 ± 16.97*	59.11 ± 4.14*	< 0.001
Inner Temporal (μm)	52.44 ± 3.57	63.17 ± 10.65*	60.44 ± 4.02*	< 0.001
Inner Inferior-temporal (μm)	52.89 ± 3.76	64.22 ± 11.82*	60.67 ± 4.73*	< 0.001
Outer Inferior (μm)	54.11 ± 3.29	57.39 ± 17.17	53.78 ± 6.45	0.780
Outer Inferior-nasal (μm)	53.78 ± 3.04	59.22 ± 15.49	52.50 ± 5.29	0.397
Outer Nasal (μm)	53.44 ± 2.75	54.67 ± 11.54	51.72 ± 6.23	0.478
Outer Superior-nasal (μm)	52.83 ± 3.67	56.00 ± 12.72	51.50 ± 5.15	0.462
Outer Superior (μm)	51.72 ± 3.34	58.00 ± 17.99	51.56 ± 5.68	0.629
Outer Superior-temporal (μm)	51.56 ± 2.87	60.44 ± 17.14	53.89 ± 6.46	0.415
Outer Temporal (μm)	51.89 ± 3.69	57.28 ± 12.30	53.67 ± 6.91	0.467
Outer Inferior-temporal (μm)	52.78 ± 3.66	57.39 ± 12.88	53.67 ± 7.63	0.871

*LASIK Xtra* Femtosecond laser-assisted in situ keratomileusis combined with intraoperative accelerated corneal collagen crosslinking, *SD* Standard deviations, *μm* Micrometer, *Pre-op* preoperative, *Post-op* Postoperative

^*^P value was statistically significant differences between postoperative and baseline

### Refractive outcomes and corneal biomechanical properties

The changes in visual acuity, corneal curvature, IOP (such as corneal compensated IOP [IOPcc] and Goldmann-correlated IOP [IOPg]), and corneal biomechanical properties (such as corneal hysteresis [CH] and corneal resistance factor [CRF]) after 12-month are summarized in Table 5. All the parameters showed statistically significant differences between the preoperative and postoperative follow-up values (*P* < 0.05 for all).

**Table 5:** Changes in UDVA, corneal curvature, IOP and corneal biomechanical parameters After LASIK Xtra

	Mean ± SD		
Parameter	Pre-op	6-month Post-op	12-month Post-op
UDVA (logMAR)	1.38 ± 0.23	0.04 ± 0.08*	0.05 ± 0.09*
Ks (D)	44.68 ± 1.51	39.24 ± 2.08*	39.36 ± 2.04*
Kf (D)	43.35 ± 1.50	38.38 ± 1.93*	38.43 ± 1.82*
AvgK (D)	44.01 ± 1.44	38.81 ± 1.99*	38.85 ± 1.96*
Cyl (D)	1.34 ± 0.85	0.86 ± 0.46*	0.92 ± 0.48*
IOPcc (mmHg)	15.46 ± 3.33	13.71 ± 2.20*	13.80 ± 1.85*
IOPg (mmHg)	13.21 ± 3.40	9.34 ± 2.16	9.37 ± 2.33*
CRF (mmHg)	8.63 ± 1.37	6.16 ± 1.26*	6.12 ± 1.38*
CH (mmHg)	9.09 ± 1.19	7.58 ± 1.21*	7.51 ± 1.12*

*LASIK Xtra* Femtosecond laser-assisted in situ keratomileusis combined with intraoperative accelerated corneal collagen crosslinking, *Pre-op* Preoperative, *Post-op* Postoperative, *UDVA* Uncorrected distance visual acuity, *Ks* Steepest keratometry, *Kf* Flattest keratometry, *AvgK* Front average keratometry, *Cyl* Keratometric astigmatism, *IOPcc* Corneal compensated intraocular pressure, *IOPg* Goldmann-correlated intraocular pressure, *CRF* Corneal resistance factor, *CH* Corneal hysteresis, *mmHg* Millimeters of mercury, *logMAR* Logarithm of the minimum angle of resolution, *D* Diopters, *mmHg* Millimeters of mercury

^*^P value was statistically significant differences between postoperative and baseline

## Discussion

Corneal surface ablation surgery has the potential to affect corneal biomechanical properties and increase the incidence of iatrogenic keratectasia when considerable corneal volume is removed or a corneal flap is created. On the contrary, the main goal of the accelerated, superficial CXL fluence is to regulate the elaborate system of epithelial-stromal communication through an increase in cytokines and growth factors to maintain corneal strength without inducing a refractive change. According to the results of the present study, significant epithelial remodeling occurred after LASIK Xtra, with a variation in epithelial profiles, fluence corneal power, and biomechanical properties compared to those observed preoperatively.

Corneal myopic ablation is the preferred tissue removal procedure for the central cornea, and it may partially account for the rarity of iatrogenic keratectasia and lower estimated postoperative CH and CRF values compared to hyperopic correction, which ablates the paracentral cornea to steepen the central cornea [16]. Therefore, the corneal biomechanical properties were weakened after refractive surgery, and this may have influenced the IOP values during the follow-up period. In the current study, IOPcc and IOPg were found to have statistically significant reductions after treatment. Flap creation during LASIK surgery causes disruption of the peripheral collagen fibers and damages the components of the extracellular matrix (ECM); therefore, ECM repair may affect CH and CRF values [17]. In this study, CH and CRF values were found to be stable after treatment.

Epithelial-stromal interactions can be mediated through soluble factors such as cytokines and growth factors or through cell–matrix interaction where the cellular behavior of the migrating cell is modified by the new environment [18]. Therefore, we hypothesized that the corneal epithelial thickness would be thickened in the early stages and remained stable after treatment for a long-term follow-up. Therefore, timely detection and monitoring of corneal epithelial profiles are becoming more important in the preclinical diagnosis of iatrogenic keratectasia [19]. During the follow-up at 12-month after the surgery, significant inner regional epithelial remodeling occurred after LASIK Xtra, and a doughnut-like profile with central thickened epithelium was observed after treatment. Kanellopoulos et al. [12] reported that the postoperative epithelial thickness increased significantly in the mid-peripheral region by 3.79 μm and 3.95 μm for the “-8.00 D to -9.00 D” and “-7.00 D to -8.00 D” after LASIK Xtra, compared to 9.75 μm and 7.14 μm after LASIK alone, for the same degree of myopia. These results confirmed the hypothesis that differences in epithelial and stromal thickness indicate corneal ECM repair after LASIK Xtra. Shih et al. [20] reported corneal stress concentration in an oblique downward direction after corneal refractive surgery. According to our results, the steepest and flattest meridian keratometry values were significantly reduced from 44.68 ± 1.51 D to 39.36 ± 2.04 D, and 43.35 ± 1.50 D to 38.43 ± 1.82 D, respectively (*P* < 0.001 for all). A similar refractive shift has been reported in another study [21].

The refractive outcomes were stable 12-month after treatment, with good results in terms of UDVA, which is consistent with the decrease in the initial corneal curvature commonly reported after LASIK Xtra [8, 21]. Kohnen et al. [8] demonstrated that postoperative UDVA significantly improved at 1 week postoperatively, but did not show significant differences between the values obtained with LASIK Xtra and LASIK, from 0.08 ± 0.14 logMAR and 0.04 ± 0.13 logMAR to -0.02 ± 0.15 logMAR and 0.01 ± 0.15 logMAR after 12-month. Eyes that underwent LASIK Xtra had an equal or better visual acuity than eyes that underwent stand-alone LASIK [8, 21], and 90% of eyes achieved 20/40 or better UDVA after LASIK Xtra in the current study.

The limitations of our study include the possible effect of a single-center retrospective clinical study, a 12-month follow-up period, small sample size of patients undergoing bilateral LASIK Xtra, and the 6-mm corneal diameter limit for epithelial remodeling. AS-OCT, which is a clinical quantitative and qualitative investigation of corneal epithelial and stromal remodeling after surgery, provides a high-quality images of the 6-mm corneal thickness profile. Kanellopoulos et al. [12] found a statistically significant reduction in epithelial increase after LASIK Xtra (30 mW/cm^2^ for a total of 80 s) compared to that after stand-alone LASIK procedures. However, flap creation and CXL are affected by central corneal thicknesses greater than 6 mm. Future cohort studies should involve larger sample sizes and allow for imaging of corneal diameters up to 10 mm, which would cover the entire area affected by LASIK flap creation and CXL. These studies can provide a greater insight into the entire area of epithelial remodeling.

## Conclusions

LASIK Xtra provided an effect equal to that of CXL on corneal anterior stromal in terms of strengthening the corneal biomechanical properties, mostly likely due to corneal anterior stromal fibroblast activity. In addition, the improvement in UDVA and corneal biomechanical stability could potentially lead to more definite changes in corneal curvature. However, detection and monitoring of epithelial remodeling profile maps could be a more sensitive measurement tool than the assessment of surface topography or stromal surface changes induced by LASIK Xtra.

## Data Availability

Data is available from the first author on reasonable request.

## Abbreviations

LASIK: Femtosecond laser-assisted in situ keratomileusis
LASIK Xtra: LASIK procedures combined with intraoperative accelerated corneal collagen crosslinking
logMAR: Logarithm of the minimum angle resolution
CXL: Corneal collagen crosslinking
UVA: Ultraviolet-A
CI: Central islands
UDVA: Uncorrected distance visual acuity
CDVA: Corrected distance visual acuity
IOP: Intraocular pressure
IOPcc: Compensated intraocular pressure
IOPg: Goldmann-correlated intraocular pressure
AS-OCT: Anterior segment optical coherence tomography
CRF: Corneal resistance factor
CH: Corneal hysteresis
ECM: Extracellular matrix
